# Sestrin2 protects dendrite cells against ferroptosis induced by sepsis

**DOI:** 10.1038/s41419-021-04122-8

**Published:** 2021-09-04

**Authors:** Jing-yan Li, Chao Ren, Li-Xue Wang, Ren-qi Yao, Ning Dong, Yao Wu, Ying-ping Tian, Yong-ming Yao

**Affiliations:** 1 Department of Emergency, The Second Hospital of Hebei Medical University, Shijiazhuang, 050000 People’s Republic of China; 2 Translational Medicine Research Center, Medical Innovation Research Division and Fourth Medical Center of the Chinese PLA General Hospital, Beijing, 100048 People’s Republic of China

**Keywords:** Cell death, Immunology

## Abstract

Ferroptosis is a nonapoptotic form of programmed cell death triggered by the accumulation of reactive oxygen species (ROS) depended on iron overload. Although most investigations focus on the relationship between ferroptosis and cancer, neurodegenerative diseases, and ischemia/reperfusion injury, research on ferroptosis induced by immune-related inflammatory diseases, especially sepsis, is scarce. Sestrin2 (Sesn2), a highly evolutionary and stress-responsive protein, is critically involved in defense against oxidative stress challenges. Upregulated expression of Sesn2 has been observed in preliminary experiments to have an antioxidative function in the context of an inflammatory response. Nevertheless, the underlying function of Sesn2 in inflammation-mediated ferroptosis in the immune system remains uncertain. The current study aimed to demonstrate the protective effect of Sesn2 on ferroptosis and even correlations with ferroptosis and the functions of ferroptotic-dendritic cells (DCs) stimulated with lipopolysaccharide (LPS). The mechanism underlying DCs protection from LPS-induced ferroptosis by Sesn2 was further explored in this study. We found that the immune response of DCs assessed by co-stimulatory phenotypes was gradually enhanced at the peak time of 12 h upon 1 μg/ml LPS stimulation while ferroptosis in DCs treated with LPS at 24 h was significantly detected. LPS-induced ferroptosis showed a suppressive impact on DCs in phenotypic maturation, which was conversely relieved by the ferroptotic inhibitor. Compared with wild-type (WT) mice, DCs in genetic defective mice of Sesn2 (Sesn2^−/−^) exhibited exacerbated ferroptosis. Furthermore, the protective effect of Sesn2 on ferroptosis was noticed to be associated with the ATF4-CHOP-CHAC1 pathway, eventually exacerbating ferroptosis by degrading of glutathione. These results indicate that Sesn2 can suppress the ferroptosis of DCs in sepsis by downregulating the ATF4-CHOP-CHAC1 signaling pathway, and it might play an antioxidative role.

## Introduction

Sepsis, resulting from complicated infection, trauma, shock, and major surgery, can lead to circulatory disturbance and multiple organ dysfunction [1–3]. As a major cause of in-hospital death in intensive care units worldwide [4–6], sepsis might eventually deteriorate in a vicious cycle of uncontrolled inflammatory reactions and an immunosuppressive state [7, 8]. Many investigations of the underlying pathophysiologic mechanisms of immunosuppression in sepsis have resulted in the identification of scientific interventions and potential therapeutic targets. Dendritic cells (DCs), which are representative of antigen-presenting effect in the immune system, are uniquely able to coordinate the innate immune response with the adaptive immune response [9, 10]. Moreover, they are capable of stimulating original T-cell proliferation and initiating an immune response by capturing, processing, and transporting to lymphoid organs [11, 12]. Emerging evidence has revealed that exhaustion and dysfunction of DCs might be responsible for the pathogenesis of sepsis, in turn contributing to the poor outcome of septic complications [13–17]. However, the potential mechanisms with regard to DC-mediated dysfunction induced by sepsis remain largely unclear.

Increasing studies have highlighted the crucial impact of ferroptosis, a newly proposed form of cell death. Ferroptosis is a necrotic-programmed cell death characterized by iron-overaccumulation and lipid peroxidation, which is triggered by reduced glutathione synthesis and inactive enzyme glutathione peroxidase 4 (GPX4) [18–20]. It has been documented that ferroptosis is inherently immunogenic and inflammatory because it releases cellular cytokines called danger-associated molecular patterns (DAMPs), which irreversibly trigger stress-exposed cells into a proinflammatory state [21]. Additionally, treatment with ferroptotic inhibitors markedly ameliorated sepsis induced by lipopolysaccharide (LPS), which was related to the reduction in proinflammatory cytokines. Thus, we speculate that ferroptosis might be involved in the occurrence, development, and even prognosis of sepsis.

Sesn2, a highly conserved and stress-inducible protein, can be activated by various stimuli [22–24]. In addition to the important impact of Sesn2 on defending immune cells against multitudinous stimuli by mediating endoplasmic reticulum stress (ERS), autophagy, and apoptosis, our previous study showed that Sesn2 expression stimulated by high mobility group box-1 protein (HMGB1), was significantly upregulated, which accordingly exerted a protective effect on DCs following septic challenge [25]. Considering that Sesn2 can profoundly affect the pathological process of ferroptosis via antioxidation, we hypothesize that Sesn2 might be critically involved in the immunomodulation of DCs to protect against ferroptosis resulting from sepsis. Thus, the current study was purposed to explore a regulatory effect of Sesn2 on ferroptosis of DCs and identify the underlying signaling pathway in the setting of sepsis.

## Results

### LPS-mediated ferroptosis and changes in co-stimulatory phenotypes of DCs

**Fig. 1:**
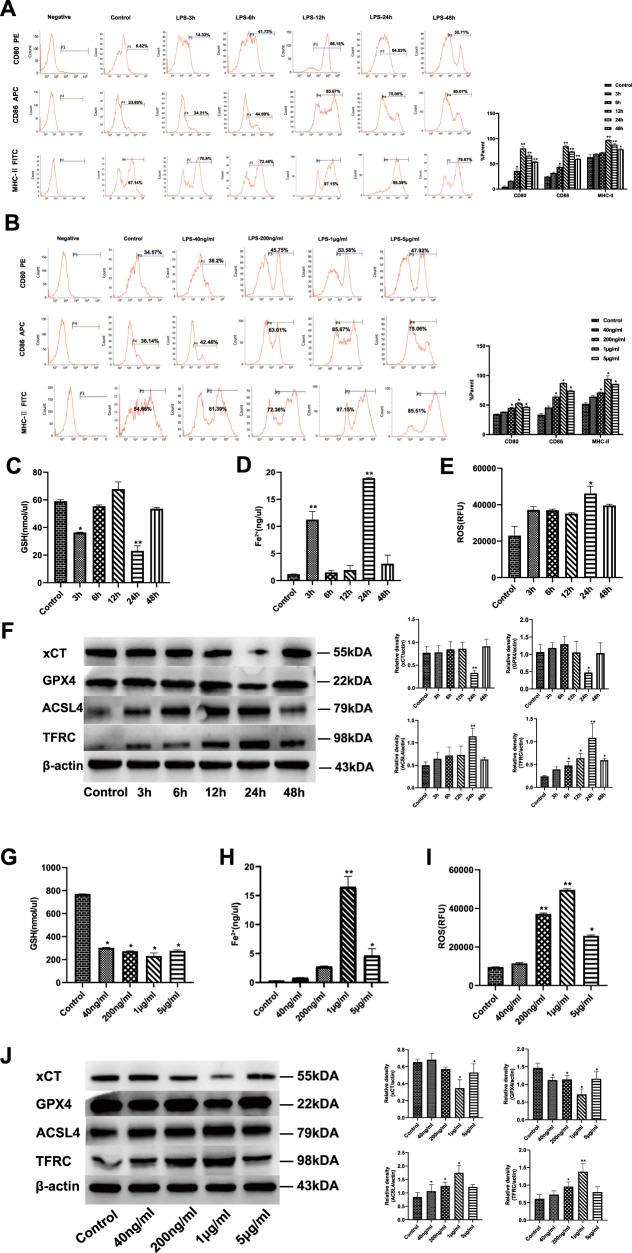
Treatment with LPS upregulates ferroptosis and the percentage of co-stimulatory phenotypes of DCs. DCs were stimulated with LPS at various concentrations (40 ng/ml, 200 ng/ml, 1 μg/ml, 5 μg/ml) for different intervals (0, 3, 6, 12, 48 h). DCs cultured without LPS were defined as controls. **A**, **B** Representative flow cytometric analysis was performed for CD80, CD86, and MHC-II expression on DCs. **C**–**E**, **G**–**I** Intracellular levels of GSH, Fe^2+^, and ROS in DCs were measured by detection kits. **F**, **J** Ferroptosis-related proteins were analyzed as presented in the methods. Data were presented as the mean ± SD of three replicates (*n* = 3 per group). Statistical significance: *P < 0.05, **P < 0.01 vs. the control group.

As illustrated in Fig. 1A, B, the immune response of DCs assessed by phenotypes was gradually enhanced with stimulation of time and dose, peaking at 12 h and a concentration of 1 μg/ml LPS, while declining after 24 h and a dose of 5 μg/ml (*P* < 0.05 or *P* < 0.01). As shown in Fig. 1C–E, G–I, compared to the peak value at 3 h, glutathione (GSH) level was significantly reduced while contents of Fe^2+^ and ROS were increased in DCs stimulated by LPS at a dose of 1 μg/ml at 24 h (*P* < 0.05 or *P* < 0.01). Stimulation with LPS (1 μg/ml at 24 h) decreased xCT and GPX4 expressions, and upregulated acyl-CoA synthetase long-chain family member 4 (ACSL4) and transferrin receptor (TFRC) expressions (Fig. 1F, J, *P* < 0.05 or *P* < 0.01).

### LPS-induced ferroptosis results in a suppressive impact on the maturational functions of DCs

**Fig. 2:**
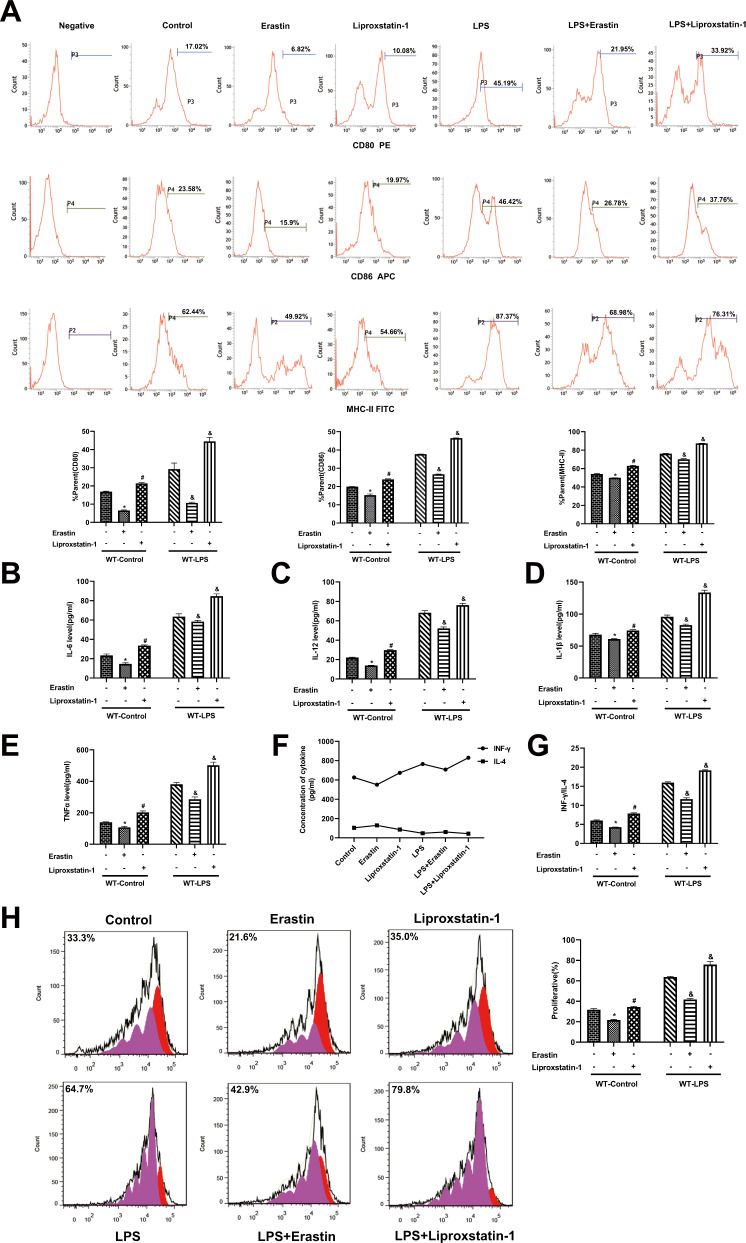
LPS-induced ferroptosis results in a negative effect on the immune function of DCs. DCs were respectively pretreated with Era (20 μM) and Lip-1 (1 μM) 1 h prior to LPS stimulation. **A** Percentages of co-stimulatory phenotypes expressed on DCs. **B**–**E** Supernatant levels of IL-12, IL-6, IL-1β, and TNF-α in cultured DCs were detected by ELISA. **F**–**G** CD4 ^+^T cells stained with Con A were incubated with DCs at a ratio of 1:200. Levels of IFN-γ and the IFN-γ/IL-4 ratio were examined by ELISA to evaluate Th1/Th2 polarization. **H** Stimulated DCs were cocultured with CD4^+^ T cells incubated with Con A, and then T-cell proliferation induced by DCs was evaluated by flow cytometry. The results were shown as the as mean ± SD (*n* = 3 in each group). Statistical significance: *P < 0.05 for the WT-Era group vs. the WT-control group; ^*#*^*P* < 0.05 for the WT-Lip-1 group vs. the WT-Era group; ^&^*P* < 0.05 vs. the WT-LPS group.

In previous study, we reported that immune function of DCs could be initiated to a certain degree but exhausted in the anaphase of LPS challenge [26, 27]. To investigate the maturation of ferroptotic-DCs after LPS stimulation, primary DCs were treated with the ferroptosis inducer Erastin (Era) (20 μM) and an inhibitor of ferroptosis, Liproxstatin-1(Lip-1) (1 μM), 1 h prior to LPS treatment. Compared with the WT-control group, phenotypic maturation and the release of cytokines were decreased in the WT-Era group and were partly recovered in the WT-Lip-1 group. The indicators in WT-LPS + Era group were significantly lower, and in the WT-LPS + Lip-1 group were higher than those in the WT-LPS group (Fig. 2A–E, *P* < 0.05).

To evaluate the maturational capacity of DC-mediated proliferation and differentiation with T cells, pretreated DCs were cocultured with T cells stimulated with concanavalin A (Con A). As shown in Fig. 2F–H, compared with the WT-control group, interferon (IFN)-γ/interleukin (IL)-4 ratio and T-cell proliferative activity were decreased in the WT-Era group, which were increased in the WT-Lip-1 group. The proliferation of Th1 and Th2 cytokines of DCs with T cells in the WT-LPS + Era group was lower whereas that in the WT-LPS + Lip-1 group was higher than that in the WT-LPS group.

### Sesn2 maintains the immune function of DCs by inhibiting LPS-induced ferroptosis

#### Sesn2 inhibits ferroptosis of DCs after LPS stimulation

**Fig. 3:**
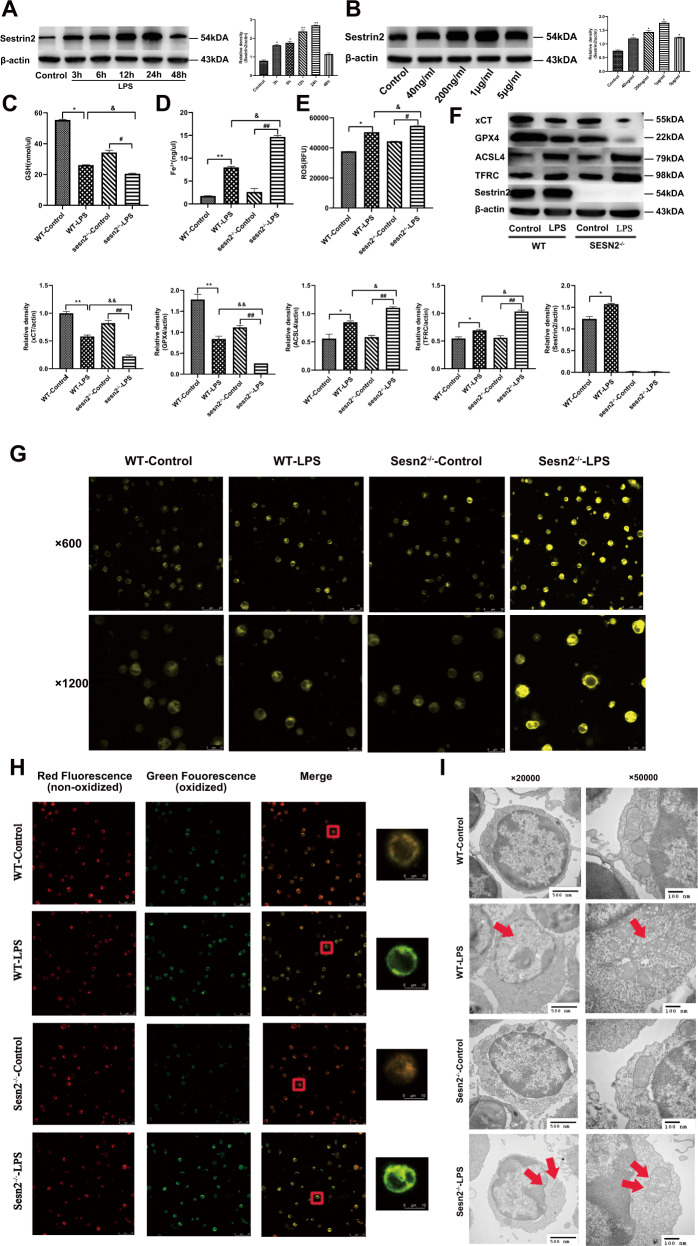
Sesn2 inhibits ferroptosis of DCs under LPS stimulation. **A**, **B** Expression of Sesn2 was analyzed by immunoblotting after DCs were stimulated with different doses (40 ng/ml, 200 ng/ml, 1 μg/ml, 5 μg/ml) of LPS for various durations (0, 3, 6, 12, 48 h). β-actin was as the base standard. **C**–**E** DCs were isolated from splenocytes of WT mice and Sesn2^−/−^ mice. The contents of GSH, Fe^2+^, and ROS in DCs were measured by detection kits. **F** Expressions of xCT, GPX4, ACSL4, TFRC, and Sesn2 were assessed by Western blotting. **G** Intracellular iron in DCs stained with FerroOrange probes was measured with fluorescence intensity by confocal laser scanning microscopy (×600 and ×1200), scale bar = 25 μm, scale bar = 10 μm. **H** Lipid peroxidation of DCs was detected by C11-BODIPY after exposure to LPS for 24 h. Red fluorescence represented nonoxidized lipids while green fluorescence was a landmark for oxidation (×600 and ×1200), scale bar = 25 μm, scale bar = 10 μm. **I** Transmission electron microscopy was applied to observe the morphology of ferroptosis in DCs exposed to LPS, which showed cytoplasmic dilatation and mitochondrial cristae dysfunction (500 nm and 100 nm). Results were shown as the mean ± SD of three replicates (*n* = 3 per group). Statistical significance: *P < 0.05, **P < 0.01 vs. the WT-control group; ^#^*P* < 0.05, ^##^*P* < 0.01 vs. the Sesn2^−/−^-control group; ^&^*P* < 0.05, ^&&^*P* < 0.01 as the Sesn2^−/−^-LPS group vs. the WT-LPS group.

As shown in Fig. 3A, B, compared with the nomal controls, Sesn2 protein expression was significantly upregulated in DCs after treatment with 1 μg/ml LPS for 24 h (*P* < 0.05 or *P* < 0.01). We treated DCs extracted from splenocytes of WT and Sesn2^−/−^, with 1 μg/ml LPS for 24 h. The GSH contents in DCs were reduced and levels of Fe^2+^ and ROS were increased. Depletion of GSH was more evident, while Fe^2+^ and ROS levels were higher in the Sesn2^−/−^-LPS group than in the WT-LPS group (Fig. 3C–E, *P* < 0.05 or *P* < 0.01). The expressions of xCT and GPX4 were decreased while ACSL4 and TFRC were upregulated in DCs of the Sesn2^−/−^-LPS group when compared with the WT-LPS group (Fig. 3F, *P* < 0.05 or *P* < 0.01).

We next examined intracellular iron content using FerroOrange probes, which stained the cells gradually with light fluorescence intensity when intracellular Fe^2+^ was generated. The images presented in Fig. 3G showed that DCs exposed to LPS exhibited iron overload, and this effect was more notable in the Sesn2^−/−^-LPS group. In addition, laser scanning confocal microscopy (LSCM) was applied to reveal the impact of LPS-induced ferroptosis on oxidative and antioxidative responses of DCs. Combined with the images shown in Fig. 3H, DCs from Sesn2^−/−^ mice exposed to LPS presented more severe oxidation than WT-LPS group.

Transmission electron microscope (TEM) was used to observe alterations in the cytomembrane, cytoplasm, and mitochondria. As shown in Fig. 3I, compared to the controls, DCs stimulated in the WT-LPS and Sesn2^−/−^-LPS groups exhibited cytoplasmic dilation instead of perinuclear dilated in addition to increased mitochondrial membrane and destroyed mitochondrial cristae, which was consistent with previous reports [28, 29].

#### Protective effect of Sesn2 on DCs is associated with the suppression of ferroptosis

**Fig. 4:**
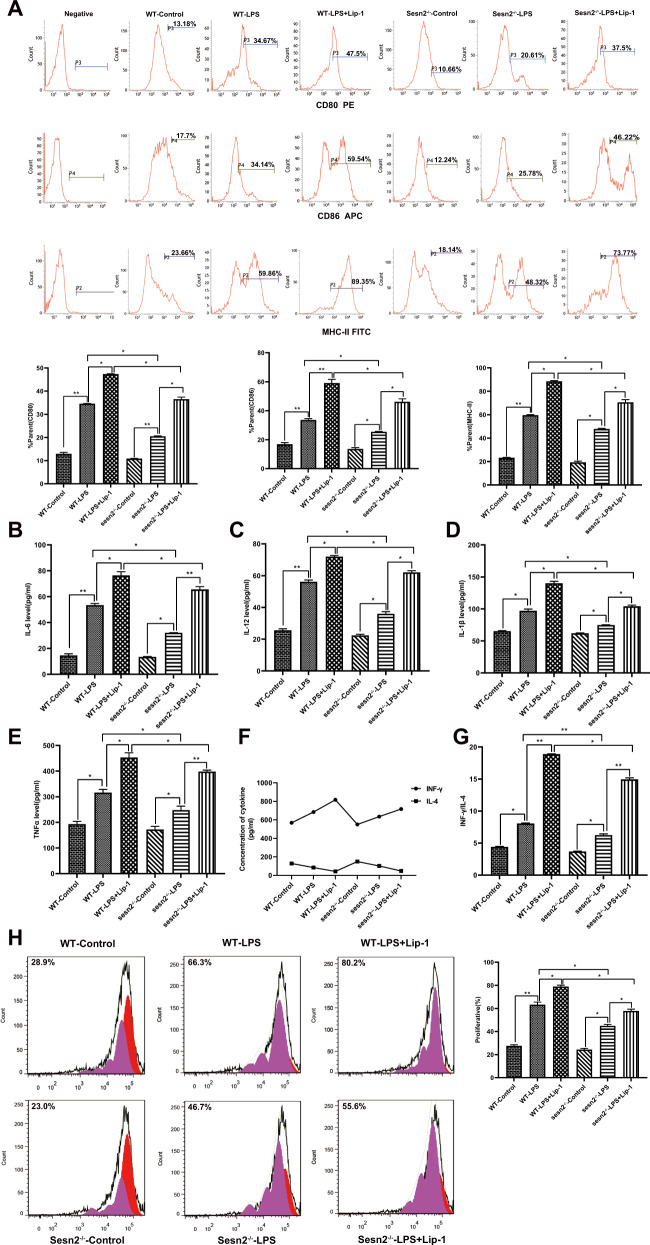
The protective effect of Sesn2 on DCs is related to the inhibition of ferroptosis. DCs obtained from splenocytes of WT mice and Sesn2^−/−^ mice, were pretreated with Lip-1 (1 μM) 1 h prior to LPS stimulation. **A** Expression of co-stimulatroy phenotypes was determined in murine DCs treated with LPS or pretreated with Lip-1 in advance of LPS. **B**–**E** Secretion levels of inflammatory cytokines in supernatants from stimulated DCs were assessed by ELISA. **F**–**G** CD4^+^ T cells incubated with Con A were cocultivated with DCs at a ratio of 1:200. Levels of IFN-γ and the IFN-γ/IL-4 ratio were evaluated by ELISA. **H** Proliferation of CD4^+^ T cells was examined by CFSE staining after stimulation for 48 h with DCs pretreated with LPS or Lip-1. Values of three independent experiments were expressed as the mean ± SD (*n* = 3 per group). Statistical significance: *P < 0.05, **P < 0.01 vs. the control group.

Prior to stimulation with LPS, we treated DCs with Lip-1 to interrupt ferroptosis. As illustrated in Fig. 4A–E, percentages of co-stimulatroy phenotypes and inflammatory cytokines in the LPS + Lip-1 groups of both WT and Sesn2^−/−^ mice were significantly increased compared with those in the LPS groups. These indicators in the Sesn2^−/−^-LPS + Lip-1 group trended a descended toward than those in the WT-LPS + Lip-1 group (*P* < 0.05 or *P* < 0.01).

DCs cocultured with T cells in both WT-LPS and Sesn2^−/−^-LPS groups, tended to exhibit increases in IFN-γ/IL-4 ratios and CD4^+^ T-cell proliferation in comparison to the controls, which were more pronounced in the LPS + Lip-1 group. The indicators in the Sesn2^−/−^-LPS + Lip-1 group were lower than those in the WT-LPS + Lip-1 group (Fig. 4F–H, *P* < 0.05 or *P* < 0.01).

### Sesn2 protects DCs from ferroptosis to improve the immune response in septic mice

#### Sesn2 inhibits the ferroptosis of DCs induced by cecal ligation and puncture (CLP)

**Fig. 5:**
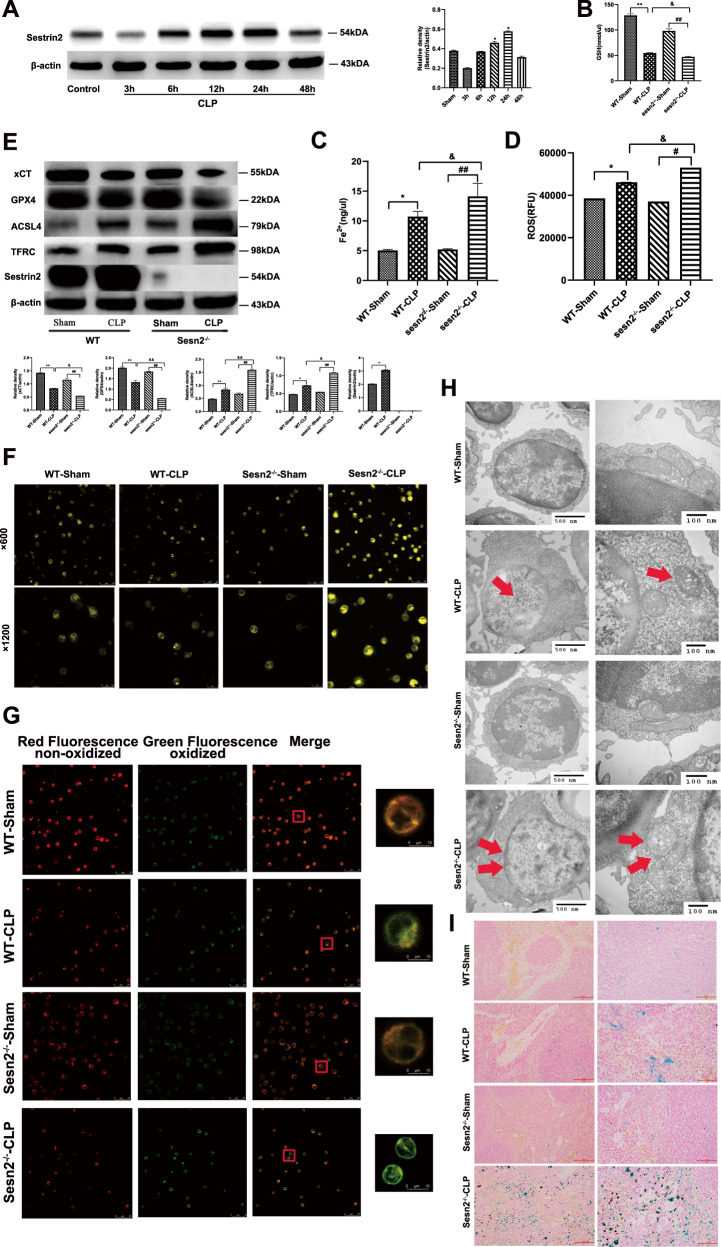
Sesn2 protects DCs from ferroptosis in septic mice with CLP. Mice underwent a CLP procedure or sham operation. **A** Protein expression of Sesn2 was detected by immunoblotting at various time points of CLP. **B**–**D** Detection kits were used to assess intracellular levels of GSH, Fe^2+^, and ROS in DCs 24 h after the operation. **E** Sesn2 and ferroptosis-associated proteins including xCT, GPX4, ACSL4, and TFRC were assessed by Western blotting after CLP. **F** Confocal laser scanning microscopy was employed to evaluate overloaded iron in DCs by fluorescence brightness labeled with FerroOrange probes (×600 and ×1200), scale bar = 25 μm, scale bar = 10 μm. **G** Ferroptosis-induced lipid peroxidation after CLP surgery for 24 h exhibited green fluorescence, while DCs in the control group were signed in red (×600 and ×1200), scale bar = 25 μm, scale bar = 10 μm. **H** Representative morphological alterations in cytoplasmic dilatation, shrunken volume of mitochondria, and mitochondrial cristae dysfunction in DCs induced by ferroptosis after CLP were examined by transmission electron microscopy (500 nm and 100 nm). **I** Iron deposition in splenic tissue of CLP mice was detected by prussian blue staining, which presented as brillant blue with a reunion distribution. The results were displayed for three repetitions as the mean ± SD. *P < 0.05, **P < 0.01 vs. the WT-control group; ^#^*P* < 0.05, ^##^*P* < 0.01 vs. the Sesn2^−/−^-control group; ^&^*P* < 0.05, ^&&^*P* < 0.01 as the Sesn2^−/−^-CLP group vs. the WT-CLP group (*n* = 3 per group).

We initially measured the expression of Sesn2 in splenic DCs extracted from septic mice at different time points, revealing peaked expression at 24 h compared with the sham group (Fig. 5A, *P* < 0.05). It showed a lower level of GSH, but higher contents of Fe^2+^ and ROS in DCs from the Sesn2^−/−^-CLP group in contrast to the WT-CLP group (Fig. 5B–D, *P* < 0.05 or *P* < 0.01). After the CLP procedure at 24 h, xCT and GPX4 levels were decreased, and expressions of ACSL4 and TFRC were enhanced. Compared with the WT-CLP group, knockout of Sesn2 resulted in downregulated expression of xCT and GPX4 but upregulated activity of ACSL4 and TFRC as shown in Fig. 5E (*P* < 0.05 or *P* < 0.01).

Twenty four hours after CLP operation, DCs stained with FerroOrange probes were detected to be more saturated than those in the sham group. In the Sesn2^−/−^-CLP group, more superfluous cellular iron was found in comparison to the WT-CLP group (Fig. 5F, shown in saffron yellow). To further investigate ferroptosis-induced lipid peroxidation 24 h after CLP, we employed LSCM to measure DCs labeled with fluorochrome of C11-BODIPY, which eventually converted nonoxidative status (shown in saffron red) into oxidative status (shown in saffron green). Imaging of DCs in the Sesn2^−/−^-CLP group revealed that more oxidative production resulted in brighter green than that observed in the WT group (Fig. 5G).

DCs in Sesn2^−/−^ mice were morphologically altered in the context of sepsis, and it was characterized by cytoplasmic dilatation, mitochondrial membrane incrassation, and mitochondrial cristae disorder (Fig. 5H). To clarify iron deposition in splenic tissue of septic mice, prussian blue staining was used to detect the expression of iron. Histomorphological alterations of ferroptosis were observed in spleens following septic challenge, which presented as brilliant blue staining and reunion distribution (Fig. 5I).

#### Sesn2 suppresses septic-induced ferroptosis to improve the immune function of DCs

**Fig. 6:**
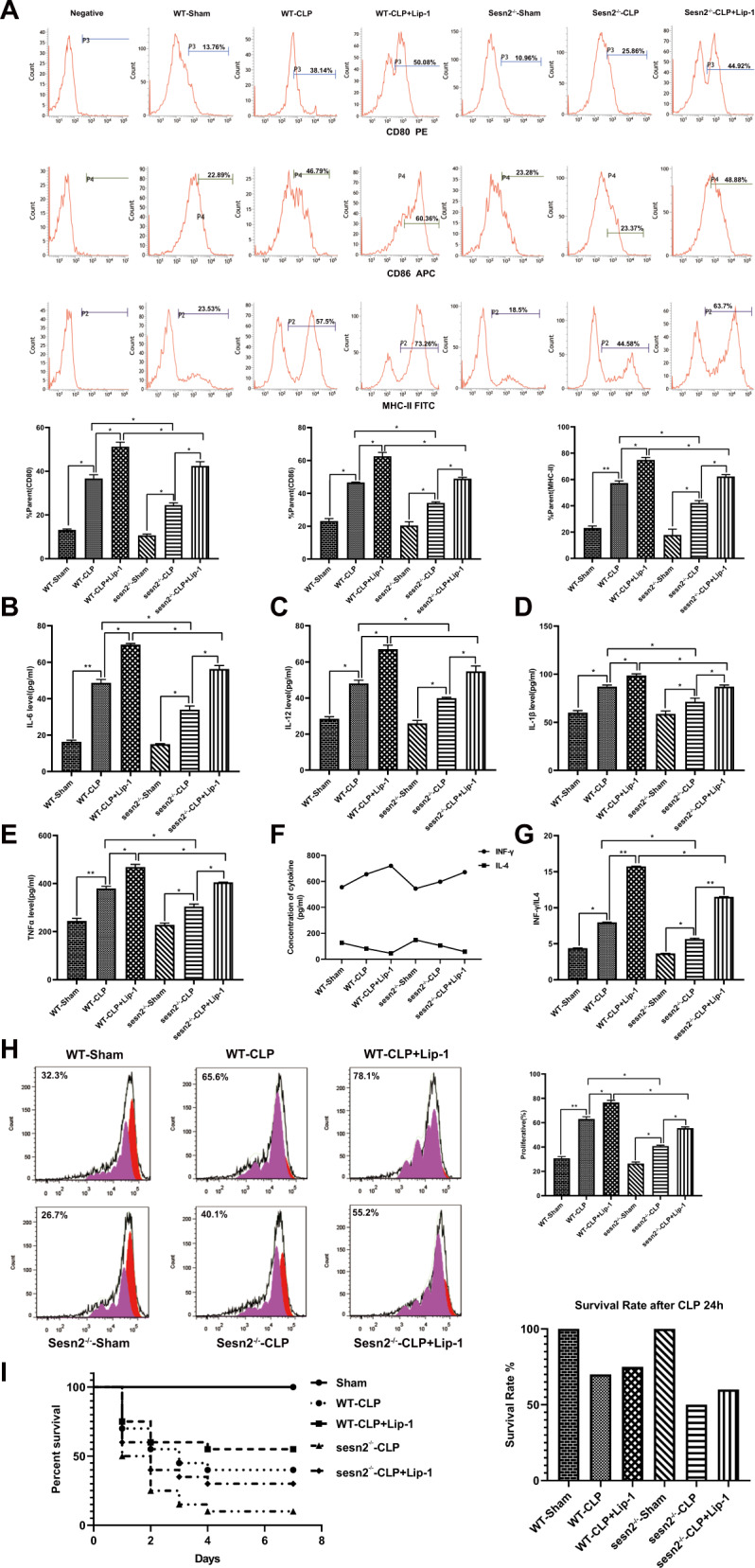
Sesn2 downregulates sepsis-induced ferroptosis to protect the immune response of DCs. Mice were intraperitoneally preinjected with Lip-1 at 10 mg/kg and underwent severe CLP 1 h later. Mice in the control group underwent a sham operation. **A** The percentage of co-stimulatory phenotypes expressed on DCs was measured by flow cytometry. **B**–**E** The release of inflammatory cytokines reflecting the degree of DC maturity and secretion was detected by ELISA after CLP. **F**, **G** DCs extracted in CLP with injection of Lip-1 or without Lip-1 were cocultured with CD4^+^ T cells incubated with Con A. Afterwards, IFN-γ contents and IFN-γ/IL-4 ratios were determined by ELISA to evaluate Th1/Th2 polarization. **H** DCs from CLP mice treated with or without Lip-1, were cocultured with CD4^+^ T cells stained with Con A, and then T-cell proliferation induced by DCs was measured by flow cytometry. **I** Mice were treated with Lip-1 at a concentration of 10 mg/kg and underwent severe CLP 1 h later. Data from three independent experiments were presented as the mean ± SD (*n* = 3 per group). Statistical significance: *P < 0.05, **P < 0.01 vs. the control group.

In the treatment group, mice were treated by intraperitoneal injection of Lip-1 (10 mg/kg) 1 h before CLP operation. As shown in Fig. 6A–E, the function of DCs in the CLP + Lip-1 groups from both WT and Sesn2^−/−^ mice, were significantly elevated compared with those in the CLP groups of WT and Sesn2^−/−^ mice (*P* < 0.05 or *P* < 0.01). As expected, compared to the controls, DCs cocultured with T cells in the WT-CLP and Sesn2^−/−^-CLP groups, tended to exhibit increased IFN-γ levels and IFN-γ/IL-4 ratios, as well as CD4^+^ T-cell proliferation, and these differences were marked in the CLP + Lip-1 group. Proliferation of DCs on T cells in the Sesn2^−/−^-CLP + Lip-1 group was obviously lower than that in the WT-CLP + Lip-1 group (Fig. 6F–H, *P* < 0.05 or *P* < 0.01).

We further compared the survival rates of Sesn2^−/−^ mice to that of WT mice at 24 h and 7 days, especially both genotypes of the CLP groups with Lip-1 injection. Sesn2 protein exerted a protective effect against CLP-induced mortality, and inversely knockout of Sesn2 exacerbated mortality, particularly at the early stage of sepsis. Additionally, it showed that CLP-induced mortality was decreased after treatment with Lip-1 (Fig. 6I).

### Sesn2 downregulates ATF4-CHOP-CHAC1 signaling pathway and protects DCs against LPS-induced ferroptosis

**Fig. 7:**
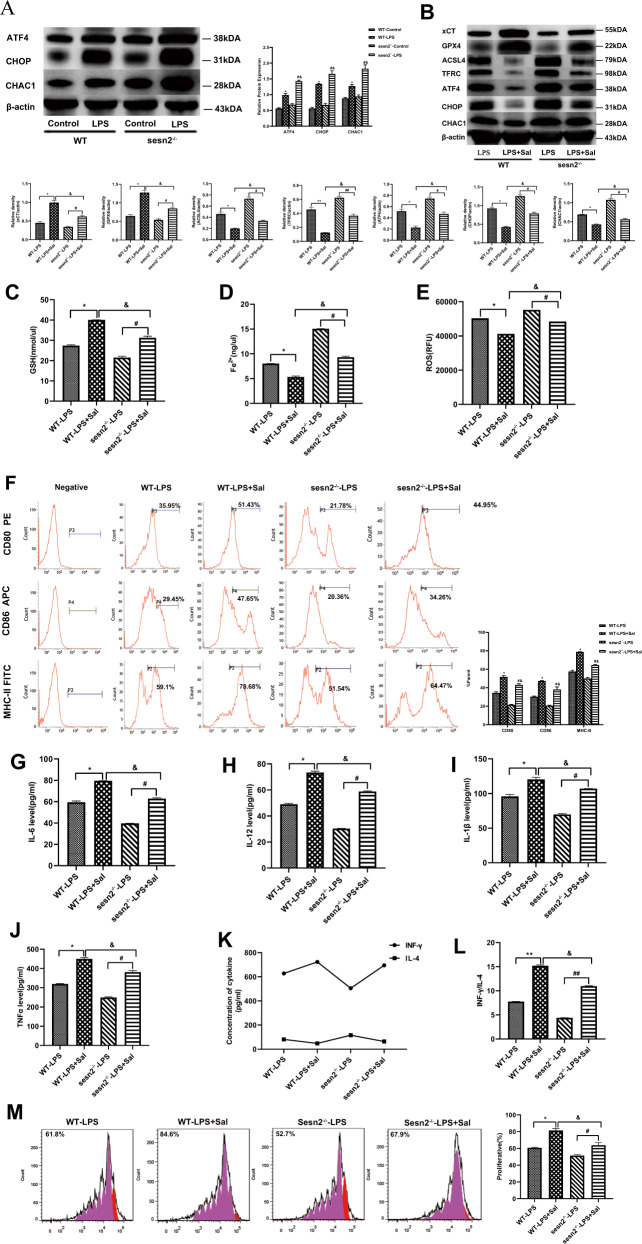
Sesn2 inhibits the ATF4-CHOP-CHAC1 signaling pathway to protect DCs against LPS-induced ferroptosis. DCs were treated with Sal (20 mM/L) 1 h before LPS stimulation. Cells cultured for 24 h with only LPS were used as the control. **A** Expressions of ATF4, CHOP, and CHAC1, as detected by Western blotting, were increased under LPS stimulation. *P < 0.05 for the WT-LPS group vs. the control group; ^#^*P* < 0.05 for the Sesn2^−/−^-LPS group vs. the control group**;** ^&^*P* < 0.05 for the Sesn2^−/−^-LPS group vs. the WT-LPS group. **B** Expressions of signaling pathway-related proteins including ATF4, CHOP, CHAC1 and the ferroptosis-related proteins xCT, GPX4, ACSL4, and TFRC in DCs pretreated with Sal before LPS stimulation, were determined by immunoblotting. **C**–**E** Levels of GSH, Fe^2+^, and ROS in DCs were measured by detection kits. **F** Percentages of costimulatory phenotypes expressed on DCs were detected by flow cytometry. **G**–**J** The release of IL-12, IL-6, IL-1β, and TNF-α, reflecting the degree of DC maturity and secretion was measured by ELISA. **K**–**L** DCs were cocultured with CD4^+^ T cells incubated with Con A to further determine the levels of IFN-γ and the IFN-γ/IL-4 ratio by ELISA. **M** Flow cytometry results. Data were presented as the mean ± SD from three replicates (*n* = 3 per group). Statistical significance: *P < 0.05, **P < 0.01 for the WT-LPS + Sal group vs. the WT-L*P*S group; ^#^*P* < 0.05, ^##^*P* < 0.01 for the Sesn2^−/−^-LPS + Sal group vs. the Sesn2^−/−^-LPS group; ^&^*P* < 0.05, ^&&^*P* < 0.01 for the Sesn2^−/−^-LPS + Sal group vs. the WT-LPS + Sal group.

As shown in our previous report that Sens2 could exert a suppressive effect on the ERS-related signaling pathway by activating transcription factor (ATF4)-C/EBP homologous protein (CHOP) to protect DCs against apoptosis [25], we speculated whether ATF4-CHOP was related to the protective impact of Sesn2 on ferroptosis in DCs. Since it has been demonstrated that activation of the ATF4-CHOP-cation transport regulator homolog 1 (CHAC1) pathway enhances ferroptosis due to the degradation of numerous glutathiones by CHAC1, we try to clarify whether Sesn2 protects DCs against ferroptosis by inhibiting the ATF4-CHOP-CHAC1 signaling pathway. The expressions of signaling related proteins including ATF4, CHOP, and CHAC1 were obviously upregulated upon LPS stimulation in the WT-LPS and Sesn2^−/−^-LPS groups compared with their expressions in the controls (Fig. 7A, *P* < 0.05 or *P* < 0.01). DCs were pretreated with salubrinal (Sal) (20 mM/L), an inhibitor of an upstream signaling pathway for 1 h, which could effectively block the action of ATF4. Then, DCs were exposed to 1 μg/ml LPS for 24 h. Pretreatment with Sal in the LPS + Sal groups of WT and Sesn2^−/−^ mice distinctly diminished the expressions of ATF4, CHOP, and CHAC1. The proteins xCT and GPX4 were increased whereas ACSL4 and TFRC were decreased compared to the LPS groups of WT and Sesn2^−/−^ mice. Blockade of ATF4 signaling significantly attenuated the positive impact of Sesn2 in Sesn2^−/−^ DCs in comparison to that in the WT group (Fig. 7B, *P* < 0.05 or *P* < 0.01). Similarly, DCs in the LPS + Sal groups of WT and Sesn2^−/−^ mice preconditioned with Sal exhibited attenuated GSH consumption, iron overload, and ROS disequilibrium in the LPS groups of WT and Sesn2^−/−^ mice (Fig. 7C–E, *P* < 0.05).

The percentages of co-stimulatory molecules and the levels of cytokines in the LPS + Sal groups of WT and Sesn2^−/−^ mice, tended to be higher than those in the LPS groups of WT and Sesn2^−/−^ mice. These indicators were significantly decreased in the Sesn2^−/−^-LPS group compared with those in the Sesn2^−/−^-LPS + Sal group (Fig. 7F–J, *P* < 0.05). Compared to the LPS groups of WT and Sesn2^−/−^ mice, DCs pretreated with Sal exhibited an increased capacity to stimulate the differentiation of CD4^+^ T cells, and the proliferation of DCs with T cells in the Sesn2^−/−^-LPS + Sal group was markedly lower than that in the WT-LPS + Sal group (Fig. 7K–M, *P* < 0.05 or *P* < 0.01).

## Discussion

Sepsis, caused by a dysfunctional immune response to infection, is now recognized as a life-threatening clinical syndrome associated with higher mortality [30]. Although the regulation of host immunity might be a potential therapy target, there is no specific medication for the management of sepsis [31, 32]. Recent studies have documented that hypofunction of leukocytes including macrophages, T lymphocytes, and DCs, which is conducive to immunosuppression, might occur in the context of sepsis. DCs classified as critical antigen-presenting cells, play a leading role in manipulation of the adaptive immune response in sepsis. Correspondingly, as shown in this study, immune function of DCs evaluated by phenotypic maturation, release of cytokines, and proliferation as well as differentiation of T cells, was significantly decreased under LPS- or CLP-induced sepsis stimulation. In addition to the alteration of the immune reaction of DCs when sepsis occurred, a previous report on splenic DCs in septic mice also revealed a significant reduction in the number of DCs [33, 34]. It has been shown that LPS, an ectogenous damage associated molecular pattern, stimulates DCs to mature via recognition of Toll-like receptors, while mature DCs upregulate the expression of phenotypic molecules, enhance the release of cytokines, and augment proliferation and differentiation on lymphocytes and T cells [35]. Our current research demonstrated the impact of LPS on the activation of DCs in a time-dependent and concentration-dependent manner. The expression of co-stimulatory molecules on DCs reached a maximum 12 h after LPS treatment at a dose of 1 μg/ml.

Currently, a newly proposed form of cell death termed ferroptosis with unique morphological, bioenergetic as well as genetic features, mainly depends on intracellular iron overload and lipid peroxidation [18, 19]. Ferroptosis results from antioxidant system malfunction leading to the recession of cellular redox homeostasis, which is characterized by GSH depletion [20]. Coincided with these findings, the current study showed that GSH levels were obviously decreased while Fe^2+^ as well as ROS contents were increased in DCs upon LPS stimulation at a dose of 1 μg/ml for 24 h. Additionally, DCs treated with LPS exhibited downregulated xCT and GPX4 expressions, and upregulated ACSL4 and TFRC expressions, which convincingly demonstrated that ferroptosis appeared to be critically involved in the inflammatory response. It is reasonable for us to speculate that ferroptosis occurs in DCs when they are stimulated by LPS in the context of excessive inflammation. Many experiments have shown that Era, an inhibitor of ferroptosis, can relieve sepsis induced by CLP or LPS in mice, which is associated with a reduced level of inflammatory cytokines such as tumor necrosis factor (TNF)-α, IL-1β, and HMGB1 [36, 37]. Thus, the regulation of the maintenance, homeostasis, and immune functions of DCs largely depends on programmed cell death in DCs. Exploration of the programmed cell death of DCs will illustrate the potential role of DCs in the maintenance of immune tolerance. The present research investigated the impact of LPS-induced ferroptosis on the immune function of DCs, thereby elucidating potential mechanism with regard to ferroptosis in regulating DC maturation, activation, and death. It was clearly revealed that the application of ferroptotic inhibitors, including Era, suppressed the expression of phenotypic molecules and decreased the levels of inflammatory mediators as well as the proliferation of T cells, which were effectively improved by Lip-1 treatment.

Recently, Sesn2 has been reported to be related to many cellular defensive responses to reduce various stresses and maintain homeostasis [38]. Herein, the expression of Sesn2 was significantly upregulated under 24 h of LPS stimulation in a dose-dependent manner. The cytoprotective effect of Sesn2, which prevents cell ferroptosis via different signaling pathways, was verified. Park et al. [39] illustrated that Sesn2 mediated by ferroptosis protected hepatocytes from liver injury via a mechanism dependent on NF-E2-related factor 2. In accordance with these findings, we proved that knockout of Sesn2 markedly augmented ferroptosis, resulting in reduction in GSH content, elevation in Fe^2+^ and ROS levels, downregulation of xCT and GPX4 whereas upregulation of ACSL4 and TFRC expressions. Similarly, Sesn2 protected mitochondria against glucose deprivation-induced metabolic stress by activating the Sesn2-AMPK signaling pathway [40]. Our data showed that in comparison to WT mice, DCs extracted from Sesn2^−/−^ mice and then subjected to both LPS stimulation and CLP, exhibited morphological alterations of mitochondria involving increased mitochondrial membranes and destroyed mitochondrial cristae, which were consistent with previous reports [40].

We further focused on signaling pathways to explore the underlying mechanism by which Sesn2 suppresses DCs from experiencing LPS-induced ferroptosis. Our previous experiments reported that Sesn2, a regulator that protected DCs from apoptosis, was relevant to ATF4-CHOP signaling and interacted with ATF4, a signaling molecule regulating ERS [25]. In the current study, we noticed that LPS-induced ferroptosis obviously upregulated expressions of ATF4 and CHOP, which were further enhanced when Sesn2 knocked out. As we known, CHAC1 has been indicated to degrade intracellular antioxidant molecules of GSH contributing to the g-glutamyl cyclotransferase activity, which can combine with GSH [41–43]. Our data indicated that LPS-induced ferroptosis could augment the activation of CHAC1. After genetic knockout of Sesn2, ferroptosis-related cell death and CHAC1 expression were distinctly aggravated. More importantly, it has been documented that the activation of CHAC1 is closely associated with the ATF4-CHOP signaling cascade in the endoplasmic reticulum [44, 45]. Accordingly, our study showed that the ATF4-CHOP-CHAC1 signaling pathway was activated in DCs treated with LPS, which was more obvious in the setting of Sesn2 knockout. In contrast, pretreatment with Sal, an inhibitor of signaling molecules in upstream of ATF4, significantly improved ferroptosis in Sesn2^−/−^ mice. Taken together, Sesn2 is determined to be a critical regulator in suppressing DCs from inflammatory-mediated ferroptosis, which appears to be related to ATF4-CHOP-CHAC1 signal transduction.

In conclusion, our results suggest that activation of Sesn2 in the regulation of the ferroptotic signaling pathway in LPS-stimulated DCs generates a protective effect on the immune function of DCs. The expression of Sesn2 can be upregulated in accordance with the stimulation time and concentration of LPS, and treatment with LPS is able to induce ferroptosis and even inhibit the maturation and activation of DCs. Knockout of Sesn2 exacerbates LPS-induced ferroptosis by aggravating the ATF4-CHOP-CHAC1 signaling cascade and distinctly deteriorates both ferroptosis and the immune response of DCs after CLP. Therefore, these findings demonstrate that Sesn2 seems to be a negative regulator of ferroptosis by downregulating ATF4-CHOP-CHAC1 in the setting of sepsis.

Nevertheless, there are several limitations in this study. First, the modulatory effect of Sesn2 on the host immune response of DCs warrant further study in the setting of sepsis. Second, the signaling pathway involved ferroptosis induced by LPS needs to be further explored to elucidate the precise signaling of ferroptosis-mediated dysfunction of DCs in the Sesn2 transgenic model. In addition, our findings obtained from murine DCs should be confirmed in human DCs to better understand in the clinical background.

## Materials and methods

### Ethics statement

All experimental manipulations were implemented consistent with criterion of the National Institutes of Health Guide for the Care and Use of Laboratory Animals and with the permission of the Scientific Investigation Board of the Chinese PLA General Hospital (No. SYXK2016-0018), Beijing, China.

### Animals

Male mice with the category of C57BL/6 J, aged six weeks old and weighted 20–25 g, were supplied by the Centre of Laboratory Animal Science, Perking Union Medical College, Beijing, China. The genetic defective mice of Sesn2 (Sesn2^−/−^) were established by Shanghai Model Biological Center, Shanghai, China. WT mice and Sesn2^−/−^ mice were housed in SPF conditions with a consistent temperature of 22–25 °C. WT and Sesn2^−/−^ mice were randomly divided into two groups, respectively: WT-sham group, WT-CLP group, Sesn2^−/−^-sham group, Sesn2^−/−^-CLP group, *n* = 3 per sham group.

### Reagents

MicroBeads of CD11c^+^ (N418) and CD4 (L3T4) were supplied by Miltenyi Biological GmbH, Bergisch Gladbach, Germany. LPS (*E.coli* O127:B8), Con A as well as detection assay of GSH (CS0260), Fe^2+^ (MAK025), and ROS (MAK143) were obtained from Sigma-Aldrich, St Louis, MO. Fluorescent antibodies were applied to flow cytometry analysis, e.g., major CD80 conjugated to phycoerythrin (PE)-Cyanine 5 was obtained from eBioscience, San Diego, CA, and CD86 conjugated to allophycocyanin (APC), as well as major histocompatibility complex (MHC)-II conjugated to fluorescein isothioctante (FITC) were purchased from Miltenyi Biotec GmbH, Bergisch Gladbach, Germany. Rabbit anti-mouse Sesn2 (ab178518), rabbit anti-mouse xCT (ab175186), rabbit anti-mouse GPX4 (ab125066), rabbit anti-mouse ACSL4 (ab155282), rabbit anti-mouse TFRC (ab84036), rabbit anti-mouse ATF4 (ab184909), mouse anti-C/EBP homologous protein (CHOP, ab11419) and rabbit anti-mouse CHAC1 monoclonal antibodies were supplied by Abcam, Cambridge, MA. Fluorescent dye used to detect ferroptosis including C11-BODIPY ^581/591^ was purchased from Invitrogen, Carlsbad, CA, while FerroGreen probes (F374) was obtained from DojinDo, Tokyo, Japan. Enzyme-linked immunosorbent assay (ELISA) kits of IL-12p40, IL-6, IL-1β, TNF-α, IL-4, and IFN-γ were supplied by Biosource, Worcester, MA. Erastin (Era) (S7242), Liproxstatin-1 (Lip-1) (S7699), and salubrinal (Sal) (S2923) were purchased from Selleckchem, Houston, TX. Supplementarily, Era is an inducer of ferroptosis, which can both bind directly with the voltage-dependent anion channel (VDAC) isoform 2 and 3 (VDAC2/3) to induce mitochondrial injury, and interfers with the cystine/glutamate antiporter (system Xc-), finally inducing ferroptosis. Lip-1 as a spiroquinoxalinamine derivative, is a inhibitor of ferroptosis, which reduces VDAC1 oligomerization to decrease the mitochondrial ROS generation. Salubrinal, a selective phosphatase inhibitor of the p-eIF2α, inhibits ERS-mediated apoptosis induced by the protein glycosylation inhibitor of tunicamycin (TM).

### Sepsis model induced by cecal ligation and puncture

Procedure of cecal ligation and puncture (CLP) was applied to construct sepsis model in mice. Mouse was supine fixed after anesthesia with chloral hydrate. The abdominal skin was sterilized and then abdominal-incision was implemented in midline of abdomen. The cecum was exposed, ligatured, and perforated with 21-gauge needle which was performed as sepsis-model. Afterwards, the cecum was replaced to enterocoelia and the incision was sutured. Complementally, 1 ml of normal saline with the concentration of 0.9% was needed to be injected subcutaneously. The standard establishment of septic model behaved lethargy, piloerection, and diarrhea in the first 6 h after the operation. Exception for ligation and puncture procedure, mice underwent the same step in the sham group.

### Isolation of splenic DCs and CD4^+^ T cells

Spleens in mice were isolated followed by being triturated with gemfree instruments. Mononuclear cells were extracted with lymphocyte isolation fluid. Then, they were magnetized by cocktails of biology-conjugated antibodies with the concentration of 10 μl per 10^7^ cells, respectively, including CD4^+^ T-cell antibody and CD11c^+^ antibody at 4 °C for 10 min. Supernatants were discarded after centrifugation at 200 g for 10 min and DCs were collected, which were cultured in a humidified incubator with the 5% CO_2_, 37 °C condition. DCs were cultured with or without LPS stimulation followed respectively by collection for immunoblotting analysis and estimation by detection assay, flow cytometry, and LSCM. Supernatants of cultured DCs were collectedly used to measure cytokines by ELISA. Levels of cytokines including IFN-γ and IL-4 in supernatants, which co-cultured DCs and CD4^+^ T cells stained with Con A, were measured by ELISA detection assay.

### Determination of ferroptosis-related markers

Intracellular levels of GSH, Fe^2+^, and ROS were respectively determined by GSH assay kit, Fe^2+^ assay kit, and ROS detection kit in accordance with the manufacturer’s protocols.

### Western blotting

Pretreated DCs (6 × 10^6^) were collected to be splitted with compound consisted of lysis buffer, protease inhibitor, and phosphatase inhibitor. Briefly, the homogenate obtained after a cycle of split on ice and vibration for 30 min, was centrifuged at the temperature of 4 °C with 14,000 rpm for 30 min. Liquid supernatant was obtained and boiled for 5 min at 95 °C followed by commixing with SDS-loading buffer. According to the standard curve, loading quantity of protein samples was measured and then loaded separately in SDS polyacrylamide gel electrophoresis (Pulilai, Beijing, China). Sample gel was electrotransferred into nitrocellulose membranes and then blocked. Specific polyclonal antibodies were incubated with the concentration of 1:1000 to determine expressions of Sesn2, xCT, GPX4, ACSL4, TFRC, ATF4, CHOP, and CHAC1, respectively. Monoclonal mouse antibody against β-actin was standard as a control of internal reference genes.

### Laser scanning confocal microscopy

Both lipid peroxidation determined by the C11-BODIPY^581/591^ and Fe^2+^ level in cells measured by FerroOrange were observed under laser scanning confocal microscopy (LSCM) (Leica, Mannheim, Germany). Pretreated cells (2 × 10^6^) were incubated with C11-BODIPY^581/591^-reactive-working fluid (10 μM) at 5% CO_2_, 37 °C in a homothermal incubator for 30 min. Incubated cells were placed on the slide and then observed by LSCM. Similarly, stimulated cells (2 × 10^6^) were stained with FerroOrange probe at the concentration of 1 μM followed by incubated at 5% CO_2_, 37 °C in a homothermal incubator for 30 min. Cells stained with fluorescence probe emerged alteration in intensity, which was detected by LSCM.

### Transmission electron microscope

Stimulated cells were obtained and fixed with 4% glutaraldehyde at the temperature of 4 °C for 1 h. Fixed samples were sliced, which was dehydrated in ethanol and then embedded in epoxy resin. Ultrathin sections were sliced by ultramicroblade followed by being stained with uranyl acetate and lead citrate. Cells were photographed by TEM (JEOL, Peabody, MA).

### Flow cytometric technology

Pretreated DCs (5 × 10^5^ per group) obtained at various intervals, subsequently were incubated with PE-conjugated IgG specific for CD80, APC-conjugated anti-mouse CD86, FITC-conjugated anti-mouse MHC-II, respectively. DCs stained with monoclonal antibodies were reacted at 4 °C for 30 min. Finally, DCs washed twice and fixed with 1% formaldehyde, then analyzed by flow cytometry (BD Biosciences, Mountain View, CA).

### Carboxyl fluorescein succinimidyl ester staining

Con A diluted with FBS was performed to a ultimate dosage of 5 μg/ml. Con A was infused into corresponding culture plates. CD4^+^ T cells were stained by carboxyl fluorescein succinimidyl ester (CFSE) (5 μM), and incubated at room temperature in dark for 20 min, which was terminated with 5-fold volume of PBS. Afterwards, CD4^+^ T cells stained with CFSE were incubated in 96-well plates with Con A for 24 h. Finally, pretreated DCs were co-cultured with CD4^+^ T cells at a proportion of 1:100 for 72 h.

### Measurement of cytokine levels

To assess the release of IL-12, IL-6, IL-1β, and TNF-α reflecting maturation of DCs, supernatants of cultured DCs were collected and measured by ELISA kits. Chromogenic reactions were discontinued by 100 μl of orthophosphoric acid. Supernatants from co-incubated DCs with CD4^+^ T cells were collected to analyze IFN-γ and IL-4 levels by ELISA according to the manufacturer’s instructions. All the data were analyzed with the method of microplate reader (Spectra MR, Dynex, Richfield, MN).

### Prussian blue staining

The splenic tissues of mice with WT and Sesn2^−/−^ underwent CLP procedures for 24 h were prepared in advance of being embedded and sliced. The section was dewaxed and stained with iron, followed by washing. The slice was immersed in nuclear solid red dye and washed, then dewatered and sealed. Each section was observed under microscope (×400).

### Statistical analysis

Data from parameters were evaluated and indicated as mean ± standard deviation (SD) for three reduplicative experiments. Student’s *t* test was appropriated to assess the significant differences between groups while variance (ANOVA) was applied to evaluate significant deviations among the groups. Data analysis was performed with SPSS and *P* values less than 0.05 or 0.01 were considered statistically significant.

## Data Availability

The datasets used and analyzed during the current study are available from corresponding author on reasonable request.

## References

[1] Ludwig KR, Hummon AB (2017). Mass spectrometry for the discovery of biomarkers of sepsis. Mol Biosyst.

[2] Belgian Outcome in Burn Injury Study Group. (2009). Development and validation of a model for prediction of mortality in patients with acute burn injury. Br J Surg.

[3] Mouri T, Kawahara H, Matsumoto T, Ishida K, Misawa T, Yanaga K (2019). Respiratory disorder at the end of surgery for peritonitis due to colorectal perforation is a critical predictor of postoperative sepsis. In Vivo.

[4] Vincent JL, Rello J, Marshall J, Silva E, Anzueto A, Martin CD (2009). International study of the prevalence and outcomes of infection in intensive care units. JAMA.

[5] Rello J, Valenzuela-Sánchez F, Ruiz-Rodriguez M, Moyano S (2017). Sepsis: a review of advances in management. Adv Ther.

[6] Coopersmith CM, Wunsch H, Fink MP, Linde-Zwirble WT, Olsen KM, Sommers MS (2012). A comparison of critical care research funding and the financial burden of critical illness in the United States. Crit Care Med.

[7] Wiersinga WJ, Leopold SJ, Cranendonk DR, Poll TVD (2014). Host innate immune response to sepsis. Virulence.

[8] Schulte W, Bernhagen J, Bucala R (2013). Cytokines in sepsis: potent immunoregulators and potential therapeutic targets: an updated view. Mediat Inflamm.

[9] Pearce EJ, Everts B (2015). Dendritic cell metabolism. Nat Rev Immunol.

[10] Said A, Weindl G (2015). Regulation of dendritic cell function in inflammation. J Immunol Res.

[11] Fan X, Liu Z, Jin H, Yan J, Liang HP (2015). Alterations of dendritic cells in sepsis: featured role in immunoparalysis. Biomed Res Int.

[12] Kumar V (2018). Dendritic cells in sepsis: potential immunoregulatory cells with therapeutic potential. Mol Immunol.

[13] Hotchkiss RS, Tinsley KW, Swanson PE, Grayson MH, Osborne DF, Wagner TH (2002). Depletion of dendritic cells, but not macrophages, in patients with sepsis. J Immunol.

[14] Tinsley KW, Grayson MH, Swanson PE, Drewry AM, Chang KC, Karl IE (2003). Sepsis induces apoptosis and profound depletion of splenic interdigitating and follicular dendritic cells. J Immunol.

[15] Gautier EL, Huby T, Saint-Charles F, Ouzilleau B, Chapman MJ, Lesnik P (2008). Enhanced dendritic cell survival attenuates lipopolysaccharide-induced immunosuppression and increases resistance to lethal endotoxic shock. J Immunol.

[16] Efron PA, Martins A, Minnich D, Tinsley K, Ungaro R, Bahjat FR (2004). Characterization of the systemic loss of dendritic cells in murine lymph nodes during polymicrobial sepsis. J Immunol.

[17] Kushwah R, Hu J (2010). Dendritic cell apoptosis: regulation of tolerance versus immunity. J Immunol.

[18] Yang WS, SriRamaratnam R, Welsch ME, Shimada K, Skouta R, Viswanathan VS (2014). Regulation of ferroptotic cancer cell death by GPX4. Cell.

[19] Kim EH, Wong SW, Martinez J (2019). Programmed necrosis and disease: we interrupt your regular programming to bring you necroinflammation. Cell Death Differ.

[20] Dixon SJ, Lemberg KM, Lamprecht MR, Skouta R, Zaitsev EM, Gleason CE (2012). Ferroptosis: an iron-dependent form of nonapoptotic cell death. Cell.

[21] Conrad M, Angeli JPF, Vandenabeele P, Stockwell BR (2016). Regulated necrosis: disease relevance and therapeutic opportunities. Nat Rev Drug Discov.

[22] Lee JH, Budanov AV, Talukdar S, Park EJ, Park HL, Park HW (2012). Maintenance of metabolic homeostasis by Sestrin2 and Sestrin3. Cell Metab.

[23] Budanov AV, Shoshani T, Faerman A, Zelin E, Kamer I, Kalinski H (2002). Identification of a novel stress-responsive gene Hi95 involved in regulation of cell viability. Oncogene.

[24] Hay N (2008). p53 strikes mTORC1 by employing sestrins. Cell Metab.

[25] Wang LX, Zhu XM, Luo YN, Wu Y, Dong N, Tong YL (2020). Sestrin2 protects dendritic cells against endoplasmic reticulum stress-related apoptosis induced by high mobility group box-1 protein. Cell Death Dis.

[26] Luan YY, Yao RQ, Tong S, Dong N, Sheng ZY, Yao YM (2016). Effect of tumor necrosis factor-α induced protein 8 like-2 on immune function of dendritic cells in mice following acute insults. Oncotarget.

[27] Luan YY, Dong N, Xie M, Xiao XZ, Yao YM (2014). The significance and regulatory mechanisms of innate immune cells in the development of sepsis. J Interferon Cytokine Res.

[28] Miyake S, Murai S, Kakuta S, Uchiyama Y, Nakano H (2020). Identification of the hallmarks of necroptosis and ferroptosis by transmission electron microscopy. Biochem Biophys Res Commun.

[29] Friedmann Angeli JP, Schneider M, Proneth B, Tyurina YY, Tyurin VA, Hammond VJ (2014). Inactivation of the ferroptosis regulator Gpx4 triggers acute renal failure in mice. Nat Cell Biol.

[30] Singer M, Deutschman CS, Seymour CW, Shankar-Hari M, Annane D, Bauer M (2016). The Third International Consensus Definitions for sepsis and septic shock (Sepsis-3). JAMA.

[31] Prescott HC, Angus DC (2018). Enhancing recovery from sepsis: a review. JAMA.

[32] Cecconi M, Evans L, Levy M, Rhodes A (2018). Sepsis and septic shock. Lancet.

[33] Guisset O, Dilhuydy MS, Thiébaut R, Lefèvre J, Camou F, Sarrat A (2007). Decrease in circulating dendritic cells predicts fatal outcome in septic shock. Intensive Care Med.

[34] Bouras M, Asehnoune K, Roquilly A (2018). Contribution of dendritic cell responses to sepsis-induced immunosuppression and to susceptibility to secondary pneumonia. Front Immunol.

[35] Cohn L, Delamarre L (2014). Dendritic cell-targeted vaccines. Front Immunol.

[36] Oh BM, Lee SJ, Park GL, Hwang YS, Lim J, Park ES (2019). Erastin inhibits septic shock and inflammatory gene expression via suppression of the NF-κB pathway. J Clin Med.

[37] Wen QR, Liu J, Kang R, Zhou BR, Tang DL (2019). The release and activity of HMGB1 in ferroptosis. Biochem Biophys Res Commun.

[38] Kim MG, Yang JH, Kim KM, Jang CH, Jung JY, Cho IJ (2015). Regulation of Toll-like receptor-mediated Sestrin2 induction by AP-1, Nrf2, and the ubiquitin-proteasome system in macrophages. Toxicol Sci.

[39] Park SJ, Cho SS, Kim KM, Yang JH, Kim JH, Jeong EH (2019). Protective effect of sestrin2 against iron overload and ferroptosis-induced liver injury. Toxicol Appl Pharmacol.

[40] Seo K, Ki SH, Shin SM (2015). Sestrin2-AMPK activation protects mitochondrial function against glucose deprivation-induced cytotoxicity. Cell Signal.

[41] Kumar A, Tikoo S, Maity S, Sengupta S, Sengupta S, Kaur A (2012). Mammalian proapoptotic factor ChaC1 and its homologues function as gamma-glutamyl cyclotransferases acting specifically on glutathione. Embo Rep..

[42] Chen MS, Wang SF, Hsu CY, Yin PH, Yeh TS, Lee HC (2017). CHAC1 degradation of glutathione enhances cystine-starvation-induced necroptosis and ferroptosis in human triple negative breast cancer cells via the GCN2-eIF2alpha-ATF4 pathway. Oncotarget.

[43] Mungrue IN, Pagnon J, Kohannim O, Gargalovic PS, Lusis AJ (2009). CHAC1/MGC4504 is a novel proapoptotic component of the unfolded protein response, downstream of the ATF4-ATF3-CHOP cascade. J Immunol.

[44] Joo JH, Ueda E, Bortner CD, Yang XP, Liao G, Jetten AM (2015). Farnesol activates the intrinsic pathway of apoptosis and the ATF4-ATF3-CHOP cascade of ER stress in human T lymphoblastic leukemia Molt4 cells. Biochem Pharmacol.

[45] Perra L, Balloy V, Foussignière T, Moissenet D, Petat H, Mungrue IN (2018). CHAC1 is differentially expressed in normal and cystic fibrosis bronchial epithelial cells and regulates the inflammatory response induced by *Pseudomonas aeruginosa*. Front Immunol.

